# Cryptogenic organizing pneumonia caused by solanine: A case report

**DOI:** 10.1097/MD.0000000000039807

**Published:** 2024-09-27

**Authors:** Linying Wang, Lvjun Zhang, Chiqing Ying, Xuehang Jin, Mingliang Ying, Hui Chen, Dan Zhu

**Affiliations:** a Department of Respiration and Critical Care Medicine, Jinhua Municipal Central Hospital, Jinhua, Zhejiang, China; b Department of Medical Imaging, Jinhua Municipal Central Hospital, Jinhua, Zhejiang, China.

**Keywords:** cryptogenic organizing pneumonia, solanine poisoning

## Abstract

**Rationale:**

Cryptogenic organizing pneumonia (COP) is a type of pneumonia with unknown cause, presenting with symptoms like dyspnea, fever, and cough. Solanine poisoning can cause symptoms like increased heart rate, rapid breathing, sore throat, diarrhea, vomiting, and fever, but there are no known cases of it causing COP.

**Patient concerns:**

A 43-year-old man had a dry cough, worse at night, with phlegm and chest tightness after eating sprouted potatoes. No history of surgeries or family medical issues.

**Diagnosis:**

Laboratory tests and metagenomic next-generation sequencing of bronchoalveolar lavage fluid from the bilateral lower lobes did not yield a definitive pathogen. Further evaluation included testing for vasculitis-associated antibodies and rheumatologic immune markers for myositis spectra to rule out connective tissue disease-associated interstitial lung disease as the etiology of organizing pneumonia. As a result, the final diagnosis was determined to be COP.

**Interventions:**

The patient received glucocorticoid therapy and oxygen therapy, and responded well to the treatment.

**Outcomes:**

On the 10th day of hospitalization, the patient was discharged with success. A follow-up chest CT conducted over a month later revealed that the lesions in both lungs had essentially resolved, with only minor residual fibrotic changes present.

**Lessons:**

Regularly monitoring disease progression is crucial for patients with solanine poisoning who have pulmonary symptoms. Promptly conducting chest CT scans and bronchoscopy is advised to check for any infections. It is also important to rule out pneumonia related to connective tissue disease-associated interstitial lung disease and provide appropriate treatment promptly.

## 1. Introduction

Organizing pneumonia (OP), previously known as bronchiolitis obliterative with organizing pneumonia,^[1,2]^ is a rare disease with clinical manifestations including cough, dyspnea, fever, and weight loss. OP can be divided into cryptogenic organizing pneumonia (COP) and secondary organizing pneumonia (SOP), the former being of unknown etiology and classified as a type of idiopathic interstitial pneumonia, while the latter is associated with various factors, including pathogenic infections, connective tissue diseases, drug effects, chemotherapy, and radiotherapy.^[3]^

Solanine is a type of glycoalkaloid (GA) derived from the metabolic products of plants such as potatoes, which can cause poisoning in humans. After potatoes sprout, the sprouts and surrounding areas have a significantly increased solanine content. Consuming potatoes with this level of solanine can result in poisoning symptoms, which often include increased heart rate, shortness of breath, sore throat, diarrhea, vomiting, and fever.^[4]^ In addition to acute pulmonary edema, there are relatively few reports on other effects of solanine poisoning on the lungs. Here we report a rare case of a middle-aged male patient who developed cryptogenic organizing pneumonia due to solanine poisoning after consuming sprouted potatoes.

## 2. Case presentation

**Figure 1.**
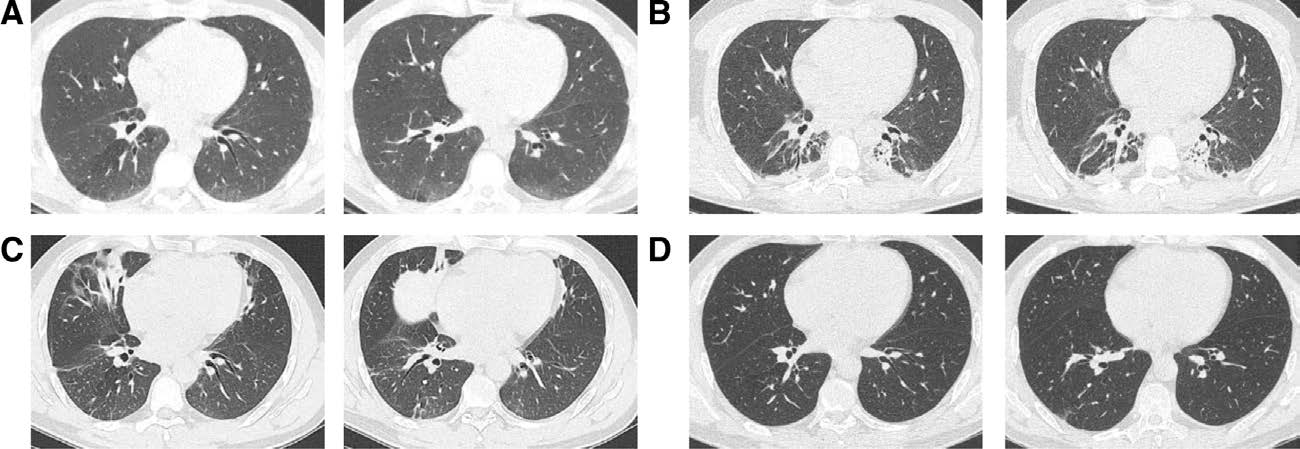
(A)The chest computed tomography scans at admission showed ground-glass opacities in the lower lobes of both lungs. (B) The patient’s condition worsened on the second day of hospitalization, with patchy high-density shadows seen in the lower lobes of both lungs. (C) The patient’s lower lung lobe lesions have been mostly absorbed, and a new lesion has developed in the right middle lung lobe. (D) One month after discharge, follow-up showed that the lesion was basically absorbed, with only a small amount of string-like high-density shadow.

The patient is a 43-year-old male who was admitted to the hospital due to cough, sputum, and chest tightness. The patient reported that he had experienced paroxysmal dry cough, which was more pronounced at night, accompanied by sputum production, a small amount of white frothy sputum, and chest tightness during severe coughing episodes. He had sought treatment at a local hospital, but after the treatment, the patient’s cough and chest tightness did not improve significantly and even worsened. He came to our hospital for further diagnosis and treatment. Upon inquiry, the patient revealed that he had consumed sprouted potatoes before the onset of the above symptoms, and denied any history of major surgery, family history, or similar episodes. Upon admission, the patient was conscious. The patient’s body temperature was 36.6 °C, pulse rate was 99 beats per minute, respiratory rate was 21 breaths per minute, and blood pressure was 121/79 mm Hg. The pulse oxygen saturation (SpO2) without oxygen supplementation was 92%, and no other apparent abnormalities were observed. Chest CT scan on admission showed a slight ground-glass opacity change in the lower lobes of both lungs, with local visible air bronchograms (Fig. 1A). Physical examination did not reveal any rash, edema, abdominal tenderness, or organ enlargement, and the extremities were free of joint deformities. After admission, immediately administer intravenous levofloxacin hydrochloride sodium chloride injection and methylprednisolone succinate sodium for treatment, after obtaining blood and sputum specimens.

Laboratory examination of the patient’s white blood cells: 9.4*10^9^/L, neutrophils 8.5*10^9^/L, CRP: 15.83 mg/L. In addition, the results of heart, liver, and kidney function showed slightly elevated levels of alanine aminotransferase, gamma-glutamyl transferase, and lactate dehydrogenase, while all other indicators were normal.

The patient’s condition began to deteriorate on the second day of admission. The patient experienced worsening chest tightness and respiratory failure with SpO2 at 88% without oxygen supplementation. The patient was immediately given 3 L/min of oxygen, but the oxygen levels did not improve significantly, so an oxygen mask (7 L/min) was used, maintaining SpO2 at 97%. Review of chest CT reveals increased inflammatory lesions in the lower lobes of both lungs compared to admission, with patchy high density shadows present (Fig. 1B).

We immediately performed bronchoscopy on the patient and obtained bronchoalveolar lavage fluid for metagenomic next-generation sequencing testing, but no definite pathogens were found. We also considered the possibility of connective tissue disease-associated interstitial lung disease (CTD-ILD) causing organizing pneumonia, but the patient’s vasculitis-related antibodies and rheumatoid immune indicators did not indicate any abnormalities. We diagnosed it as COP. The treatment was switched to intravenous injection of methylprednisolone sodium succinate 40 mg, every 8 hours for anti-inflammatory therapy for 3 days. The patient’s chest tightness and shortness of breath improved significantly, with oxygen inhalation at a rate of 3 L/min and a SpO2 of 97%. Chest CT showed obvious absorption of the bilateral pulmonary infiltrates, and a newly developed lesion in the right middle lobe (Fig. 1C). Due to a good response to treatment, the methylprednisolone dosage was gradually reduced. On the 10th day of hospitalization, the patient was discharged smoothly. Follow-up chest CT after >1 month showed basic absorption of the bilateral lesions, with only a small amount of fibroproliferative changes remaining (Fig. 1D).

## 3. Discussion

Solanine is a type of GA derived from the metabolism of plants such as potatoes, composed of solanidine as the glycoside moiety. It can be divided into solanine and chaconine based on the different connected sugar bases. The entire tuber of a potato contains GAs, with the highest content found in sprouts and skin. Among them, α-solanine and α-chaconine account for 95% of the total solanine content in potatoes and are highly toxic.^[5]^ Acute symptoms generally occur within 30 minutes to 12 hours after consumption, including nausea, vomiting, stomach cramps, abdominal pain, and diarrhea. Ingesting a dose ≥3 mg·kg can be fatal. Solanine is hardly hydrolyzed in the gastrointestinal tract and exists in the plasma in unchanged form.^[6]^ In animal experiments, it has been reported that the toxic component α-solanine can cause acute pulmonary edema,^[7]^ primarily present in the spleen, kidneys, liver, and lungs, leading to respiratory depression in poisoned rabbits.^[8]^

OP was initially described by Davison et al in 1983.^[9]^ In 1985, Epler et al described the same entity as “bronchiolitis obliterans organizing pneumonia”.^[10]^ Now, in order to avoid confusion with bronchiolitis obliterans, the preferred term is OP. One mainstream viewpoint considers OP as a nonspecific inflammatory response of the body to acute lung injury, one of which is COP, classified as idiopathic interstitial pneumonia, with unclear specific etiology. COP typically presents as subacute onset, with symptoms of dyspnea, fever, cough, and patchy infiltrates in the periphery of chest radiographs, defined histopathologically as granulation tissue plugs in small airways, bronchioles, and alveolar spaces. Another type is SOP associated with different clinical factors, such as infection (bacterial, viral, fungal, or parasitic), medications (antibiotics, antiepileptic drugs, immunomodulators), connective tissue diseases, vasculitis, or lung/bone marrow transplantation. COP and SOP have no apparent differences in terms of symptoms, signs, laboratory and pulmonary function tests, imaging, or histomorphology. Both are prone to delayed diagnosis or misdiagnosis,^[11]^ with the latter often having a higher mortality rate.^[12]^

The previously reported cases of solanine poisoning often involved strong attack and corrosion on the gastrointestinal mucosa, as well as paralytic effects on the central nervous system.^[13]^ This case report described solanine poisoning leading to COP for the first time. We conducted a differential diagnosis for this patient. The chest CT on admission showed mild ground-glass opacities, and the condition rapidly progressed on the second day, with patchy high-density shadows in the lower lobes of both lungs and respiratory failure. Routine laboratory examinations and metagenomic next-generation sequencing testing of bronchoalveolar lavage fluid did not indicate any specific pathogens, which does not support the diagnosis of community-acquired pneumonia. Vasculitis-related antibodies and rheumatoid autoimmune indicators also ruled out pneumonia caused by CTD-ILD. The imaging manifestations of COP are diverse, usually presenting as unilateral or bilateral patchy airspace consolidation with subpleural and peribronchial distribution.^[14,15]^ After excluding other factors, based on the patient’s clinical and imaging features, we considered the diagnosis of COP, with solanine poisoning from ingesting sprouted potatoes as the direct cause. Furthermore, we also found elevated alanine aminotransferase and gamma-glutamyl transferase in the patient, which may be due to the specific accumulation of α-chaconine in the liver tissue.^[16]^ This report also has several limitations, as we did not detect the levels of α-solanine, α-chaconine, and their metabolites in the patient’s blood or urine, and did not clarify whether there were other contaminants during the consumption of sprouted potatoes.

Currently, systemic corticosteroids are the main treatment for COP. Antibiotic treatment is ineffective for COP.^[11]^ About 70% to 80% of COP patients achieve complete clinical and radiological resolution with corticosteroid therapy,^[17]^ without significant sequelae.^[18]^ Due to the lack of effective antidote for solanine, we primarily use oxygen therapy and corticosteroid treatment to treat patients, and have observed promising therapeutic effects. In conclusion, we report a case of rare patient, who developed COP caused by solanine poisoning. For patients with solanine poisoning, if there are symptoms in the lungs, closely monitor the progress of the condition, and promptly undergo a chest CT scan and bronchoscopy examination to determine if there is a coexisting infection. It is also necessary to exclude pneumonia caused by CTD-ILD and provide timely treatment.

## Author contributions

**Conceptualization:** Linying Wang.

**Data curation:** Mingliang Ying.

**Formal analysis:** Chiqing Ying, Xuehang Jin.

**Investigation:** Hui Chen, Dan Zhu

**Resources:** Linying Wang, Lvjun Zhang.

**Writing – original draft:** Linying Wang, Lvjun Zhang.

**Writing – review & editing:** Hui Chen, Dan Zhu.
